# Transcultural differences of psychologically traumatised children and adolescents

**DOI:** 10.1007/s40211-019-0300-y

**Published:** 2019-02-01

**Authors:** Amesh K. Shrestha, Zeliha Özlü-Erkilic, Christian Popow, Susanne Ohmann, Türkan Akkaya-Kalayci

**Affiliations:** 1 Department of Urology, General Hospital of Feldkirch, Carinagasse 47, 6807 Feldkirch, Austria; 2 Outpatient Clinic of Transcultural Psychiatry and Migration-Induced Disorders in Childhood and Adolescence, Department of Child and Adolescent Psychiatry, Medical University of Vienna, Währinger Gürtel 18–20, 1090 Vienna, Austria; 3 Department of Child and Adolescent Psychiatry, Medical University of Vienna, Währinger Gürtel 18–20, 1090 Vienna, Austria

**Keywords:** Psychological trauma, Migration, Transcultural psychiatry, Austrian, Turkish-speaking, children and adolescents, Psychisches Trauma, Migration, Transkulturelle Psychiatrie, Österreichische, türkischsprachige Kinder und Jugendliche

## Abstract

**Background:**

The symptoms following a traumatic event as well as the coping strategies can be culture specific. The objective of the present study was to analyse the transcultural differences of psychologically traumatized children and adolescents with and without migration background.

**Methods:**

The medical files of 199 psychologically traumatized children and adolescents (99 native Austrian, 100 Turkish-speaking) who were treated at the Department of Child and Adolescent Psychiatry in Vienna were retrospectively analysed.

**Results:**

The most frequently observed causes of trauma in patients with Turkish migration background were intra-familial conflicts, forced separation from parent(s), and conforming to a new environment. In native Austrian patients, forced separation from parent(s) and divorce or separation of parents were the leading causes of trauma. Trauma-related symptoms like changed mood, cognitive and perceptual disturbance, social withdrawal, sleeping problems, somatisation and behavioural problems were similarly observed in both groups; “sleeping problems” were more often observed in Austrian patients, and “behavioural problems” were more often observed in Turkish patients. More Austrian patients (32.7%) obtained psychiatric and psychotherapeutic treatment. Turkish-speaking patients mostly obtained psychiatric treatment only. Patients with migration background were more compliant compared to indigenous patients.

**Conclusions:**

Both study groups differed in type, causes and symptoms of trauma, and in preferred therapy. Turkish-speaking patients were more compliant with therapy, as they received culture and language-sensitive medical advice and treatment in their mother tongue. Considering the cultural background of patients can optimize service delivery and therapy outcomes among children and adolescents with stressful and traumatic experiences.

## Zusammenfassung

**Hintergrund:**

Nach einem traumatischen Ereignis können sich Symptome und Bewältigungsstrategien von Kultur zu Kultur unterscheiden. Ziel der klinischen Studie war es, psychisch traumatisierte Kinder und Jugendliche mit und ohne Migrationshintergrund bezüglich transkultureller Unterschiede zu untersuchen.

**Methodik:**

Krankenakten von 199 psychisch traumatisierten Kindern und Jugendlichen (99 einheimische Österreicher, 100 türkischsprachige Migranten), die an der Universitätsklinik für Kinder- und Jugendpsychiatrie in Wien in Behandlung waren, wurden retrospektiv analysiert.

**Ergebnisse:**

Die am häufigsten beobachteten Ursachen für ein Trauma waren bei den Patienten mit türkischem Migrationshintergrund intrafamiliäre Konflikte, unfreiwillige Trennung von einem/beiden Elternteilen sowie Anpassung an eine neue Umgebung, bei den österreichischen unfreiwillige Trennung von den Eltern und Scheidung oder Trennung der Eltern. Die beiden Gruppen unterschieden sich nicht hinsichtlich der Häufigkeit folgender traumabezogener Symptome: Stimmungsschwankungen, kognitive- und Wahrnehmungsstörungen, sozialer Rückzug und somatoforme Beschwerden. Bei österreichischen Patienten wurden mehr Schlafstörungen beobachtet, bei türkischsprachigen mehr Verhaltensprobleme. Während etwa ein Drittel (32,7 %) der österreichischen Patienten psychiatrische und psychotherapeutische Behandlung erhielten, wurden türkischsprachige Patienten meist nur psychiatrisch behandelt. Patienten mit Migrationshintergrund zeigten im Vergleich zu einheimischen eine höhere Compliance mit der Behandlung.

**Schlussfolgerungen:**

Beide Studiengruppen unterschieden sich in Art, Ursachen und Symptomen des Traumas und in der bevorzugten Therapie. Türkischsprachige Patienten zeigten mehr Therapiecompliance, da sie kultur- und sprachsensible ärztliche Beratung und Behandlung in ihrer Muttersprache erhielten. Durch die Berücksichtigung des kulturellen Hintergrunds von Patienten kann die medizinische Versorgung bei traumatisierten Kindern und Jugendlichen mit Migrationshintergrund optimiert werden.

## Introduction

Psychological trauma in childhood may increase the risk of mental disorders in childhood and in adolescence [1] and may compromise healthy development [2]. According to the International Classification of Diseases(ICD)-10, psychological trauma is defined as a distressing event/situation of shorter or longer duration leading to exceptional threat or catastrophic extent and causing deep despair in almost everyone [3]. However not everyone develops a mental disorder after a traumatic event. Different factors like severity of trauma, subjective perception of trauma, type and extent of trauma, acute stress disorder, social context, premorbid experiences including pre-existing anxiety or depressive disorders and also the number of modifying factors like age, gender, economic status, and having protective factors may play a role in developing a mental disorder after a traumatic event [4–7].

Adverse and traumatic experiences in childhood may have pronounced effects on mental and physical health in adulthood [8, 9]. Furthermore, adverse or traumatic experiences in childhood mostly had a biological impact on perturbations and disease in adult life [10–12].

### Types of trauma: type I and II trauma

According to Terr (1991), traumatic events can be divided into two types. Type I trauma can be defined as a single traumatic event like natural accidents, disasters, acts of violence, kidnapping, painful medical procedures or surgery, abandonment, etc. [1]. As Terr defined type I trauma as a unique and unpredictable event, so-called mono-traumatic happenings, “to be abandoned” therefore has also been subsumed to type I trauma, although it might be debated if the prolonged consequences of this specific event would also justify classifying it as a type II trauma [13]. Type II trauma can be defined as chronic and repeated traumatic events like emotional, physical, or sexual abuse, experiencing or witnessing domestic violence, war, flight, or torture, severe bullying, etc. [1].

### Single-incidence trauma and complex trauma

The classification of trauma into type I and type II according to Terr (1991) is gradually becoming obsolete. Currently, the terms “single-incidence trauma”, which is comparable with “type I trauma”, and “complex trauma”, which is equivalent to “type II trauma”, are more commonly used. The “single-incidence trauma” is a one-off, unexpected event, e. g. death, natural disaster, rape, fire, accident [14]. Complex trauma, which is a multi-determined construct, includes child maltreatment, neglect, domestic violence and other attachment-related trauma [15–17]. It arises predominantly in early childhood or adolescence and can be defined as experiences of cumulative, chronic and prolonged traumatic events, most often of an interpersonal nature, involving primary caregivers [16, 17]. Severe neglect, exposure to domestic violence, intensive, painful medical conditions, and physical and sexual abuse can be defined as maltreatment experiences [18].

### Natural and human-made disasters

Also natural and human-made disasters can cause trauma and stress-related disorders. Natural disasters are catastrophic happenings due to natural causes such as earthquake, flooding, storms, droughts, tsunamis, volcanic eruptions etc. In contrary, human-made disasters are economic crises, terrorism, riots, war etc. [19].

### Stress-related disorders

The stress related disorders are post-traumatic stress disorder (PTSD), acute stress disorder (ASD) and adjustment disorders.

In 1992, the World Health Organisation (WHO) defined PTSD as a disorder, which people may develop in response to one or more traumatic experience(s). If the symptom duration after the traumatic experience(s) is at least one month or longer, it can be diagnosed as PTSD [20]. The severity (kind) and extent (duration) of the traumatic events play an important role in developing PTSD [4].

In contrary, by ASD the symptoms after the traumatic experience(s) last from three days to one month. People may develop ASD after an individual experience or witnessing an event involving a threat or actual death [21].

Adjustment disorder can be caused by a single event or multiple events and it is a reaction to an identifiable life stressor. People who suffer adjustment disorder are mostly impaired in their social, occupational or academic functioning. Symptoms of the adjustment disorder must arise within three months after the life event(s) happened and last no longer than six months after the stressor has ended [22].

Adjusting to a new environment or being raped or abandoned are traumatic events, which have a different scale of intensity but the individual’s reaction to the traumatic experience is also important. Many people, who underwent traumatic experience do not develop stress related disorders, they show resilience toward stressful and traumatic situations. However, children undergoing stressful events may possess fewer coping strategies than adults. Situations which seem tolerable for adults may be unduly burdening for children. Therefore the risk of developing a PTSD following traumatic events is increased in children and adolescents. Furthermore PTSD increases the risk of poor school performance, anti-social, violent or self-injuring behaviour among children and adolescents [23]. Nevertheless traumatized persons who are socially supported after the traumatic event will stabilize and recover more rapidly [7].

### Trauma and migration

With the increasing mobility in the world [24], voluntary and forced migration have progressively risen worldwide [25]. Worldwide, an incredibly large number of children and adolescents have to live separated from their parents following forced migration, which actually means a severe traumatic life-event for them [26, 27]. Refugee and migrant children experience much greater psychological distress than their native peers [28, 29] and more frequently suffer from PTSD. Therefore, children and adolescents with migration background can be defined as a vulnerable group for developing mental health problems [30] and psychiatric disorders [31–36].

### Mental health problems of migrants

Because of cultural and religious differences and issues of inequality between the country of origin and the host country, intra-familial conflicts are increased in the migrant population. These conflicts often lead to mental overload, especially for young people [37, 38]. Derluyn et al. [39] described migrant adolescents as being traumatized and showing peer problems and avoidance behaviours more frequently than their native Belgian peers. Furthermore, social, economic, and environmental problems related to the integration process will affect the mental health of migrants [40]. Specific migration related difficulties like the experience of being foreign and discriminated against racist attitudes of the host population and insufficient language skills may aggravate the acculturation process [41, 42]. Migration also affects the somatic well-being of individuals [40]. Mediterranean and non-Western cultures commonly report more somatic symptoms than psychological symptoms [43]. Valdes-Stauber et al. [44] found psychosomatic complaints in half of mentally ill Spanish migrants living in Germany.

### Aim of the present study

Trauma is a subjective experience; therefore, the reaction to a traumatic experience depends not only on severity and duration of trauma, but also on individual characteristics, such as gender, age, religious and cultural background, socio-economic status of the traumatized person. However, in Austria there are still no transcultural studies conducted with migrant children and adolescents focusing on their psychological problems following traumatic experiences. This study aims to describe transcultural differences in psychiatric diagnosis, symptoms, presentation, reasons and type of trauma, preferred type of treatment and compliance with treatment among native Austrians and Turkish-speaking children and adolescents, as Turkish-speaking migrants are the second largest group among the “third-member countries” in Austria. We assume that our study will deliver important evidence concerning transcultural differences among traumatised children and adolescents.

## Methods

We analysed the data of the 200 patients aged between 4–18 years who were treated at the Out-Patient Clinic of the Department of Child and Adolescent Psychiatry of Medical University of Vienna between 01/2009 and 12/2011 with the diagnosis of trauma-related disorder. In all, 100 patients were Austrians without migration background; 100 had a Turkish migration background. The medical record of one Austrian patient was not complete; therefore, it was excluded from the study. By using a self-developed questionnaire, we retrospectively collected socio-demographic characteristics from the patient records such as gender, age, family, living and school situation, German-language skills, integration into the peer group.

Children and adolescents originating from Turkey are after the Serbian, Bosnian or Croatian migrants the second major group of patients with migration background, from “third-member countries”, living in Austria and present at our clinic.

As the majority of the Turkish-speaking migrants came as “guest workers” to Austria, they mostly migrated voluntarily. However for the present study group there was no information whether their migration was forced or voluntary.

We investigated patients with a history of trauma-related disorders and retrospectively compared the data of patients with migration background with those of native Austrian patients. We analysed background and causes of trauma, psychological and somatic symptoms, and preferred type of therapy, patient compliance, admission ways, and factors supporting relief of trauma.

Austrian patients in this study were defined as native Austrians possessing Austrian citizenship, and having German mother-tongue. Turkish-speaking patients had parents or grandparents with a Turkish migration background. As most of the Turkish-speaking patients were born in Austria, they belong to the second or third generation migrants; therefore we preferred the expression “minors with migration background”. Among Turkish-speaking patients, the migration background was defined due to the origin of their parents/grandparents and having Turkish as mother-tongue.

Compliance with treatment means that children and adolescents regularly attended all appointments and adhered to the therapeutic recommendations.

The present study was based on a retrospective analysis of medical files. The symptoms were partly reported by the youths themselves and partly by their parent(s). The physicians have to fill out a medical-history sheet with the patients and their parent(s), in which the required aspects for their diagnostic conclusions are explicitly addressed. The attending physicians did not always note the source of the information and come to their diagnostic conclusions following the ICD-10 classification of disorders. However, the causes of trauma were mostly self-reported by the patients.

We collected retrospectively diverse socio-demographic and socio-economic characteristics like education of the patients, number of siblings and having an own room etc. but we focused on characteristics, which were relevant for studying transcultural differences among traumatised children and adolescents. Therefore, we analysed following features in the present study: age, gender, origin, type of trauma, causes of stress-related disorders, psychiatric diagnoses and stress-related symptoms.

Data were analysed using IBM SPSS, Version 21.0 (SPSS Inc., Chicago, IL, USA) software. We calculated descriptive statistics. Differences between groups were analysed by Pearson’s chi-square or Fisher’s exact test as appropriate. Statistical significance was assumed at an alpha of 5%. For comparing categorical data, we used the Mann–Whitney U‑test.

The study protocol was approved by the Ethics Committee of the Medical University of Vienna (EK number 1006/2012).

## Results

The majority (66%) of the study sample were female. About 68% of the Austrian patients and about 65% of the Turkish-speaking patients were female.

There were no significant differences concerning the age distribution of the two study groups, but still variances exist. The mean age of the Austrian children and adolescents was slightly higher (12.28) compared to Turkish-speaking (11.94) patients.

The majority (*N* = 86) of the study sample were aged between 15–18 years (43.7%), whereas only seven patients were aged between 4–6 years (3.6%), 32 patients were aged between 7–10 years old (16.2%) and 72 patients were aged between 11–14 years old (36.5%). In two cases the information about age was missing. As the numbers of patients in the age ranges were quite different and small, a comparison of the groups was not statistically meaningful. While the majority of the native Austrian patients (52.6%) were aged between 15–18 years old, the majority of the Turkish-speaking patients (39%) were aged between 11–14 years old (Table 1).

**Table 1:** Study sample

	Austrian patients (*N* = 99)		Turkish-speaking patients (*N* = 100)		Total (*N* = 199)	
	*N*	%	*N*	%	*N*	%
Female	67	67.7	65	65	132	66.3
Male	32	32.3	35	35	67	33.7
Aged between 4–6 years	2	2.1	5	5	7	3.6
Aged between 7–10 years	11	11.3	21	21	32	16.2
Aged between 11–14 years	33	34	39	39	72	36.5
Aged between 15–18 years	51	52.6	35	35	86	43.7

Significantly more Turkish-speaking than Austrian patients were presented in our out-patient clinic (*p* < 0.000) by their families. While 51.5% of Turkish-speaking patients were admitted by their families to the psychiatric outpatient clinic, only 35.4% of the native Austrians patients were referred by their families.

Significantly, more Turkish-speaking patients (93%) lived with their family compared to native Austrian patients (73%; *p* = 0.031). On the other hand, significantly, more parents of the Austrian patients were divorced, compared to Turkish-speaking patients (24%; *p* < 0.000).

Moreover, no differences between the study groups were shown concerning socio-demographic characteristics like “school situation” and “German language skills”. However, Turkish-speaking patients were better integrated into the peer group in private life compared to natives (*p* < 0.036).

### Type of trauma

Significantly more Turkish-speaking patients suffered from single trauma, whereas more Austrians suffered from chronic and repetitive trauma (*p* = 0.002, Table 2).

**Table 2:** Types of trauma

Trauma types	Austrian patients (%)	Turkish-speaking patients (%)
Single trauma	10 (10.3)	27 (27.8)
Chronic and repetitive trauma	87 (89.5)	70 (72.2)
Data missing	3	3

Pearson’s chi-square test *p* = 0.002, df = 1; Fisher’s exact test: *p* = 0.003

### Causes of stress-related disorders

Austrian and Turkish-speaking patients were significantly different with regard to causes of stressors. Divorce and separation of parents as well as separation of children from parents, maltreatment, neglect and bullying were significantly more often among the Austrian study sample compared to the Turkish-speaking sample. On the other hand, Turkish-speaking patients had more stressors due to “new environment” and “conflicts within family” than natives, but the difference was not significant (Table 3).

**Table 3:** Causes of stress-related disorders

Causes of stress-related disorders	Frequency (%)		Pearson’s χ^2^-test or Fisher’s exact test (*p* =)
	Austrian patients	Turkish-speaking patients	
Parent or grandparent death	20 (20)	15 (15)	0.352
Parents’ divorce	55 (55)	28 (28)	0.000
Parents’ separation	59 (59.6)	28 (28)	0.000
Patient’s separation from parent(s)	78 (83.9)	45 (45)	0.000
Rape	5 (5)	1 (1)	0.212
Maltreatment in childhood	13 (13)	4 (4)	0.022
Sexual abuse	7 (7)	5 (5)	0.552
Neglect	20 (20)	9 (9)	0.027
New environment	26 (26)	29 (29)	0.635
Conflict in family	40 (40)	42 (42)	0.774
Bullying	17 (17)	7 (7)	0.030

### Psychiatric diagnoses according to ICD-10

Austrian and Turkish-speaking patients had different psychiatric diagnoses. While 80 (87%) of the Austrian patients had an ICD-10 diagnosis F40–49 (anxiety, dissociative, stress-related, somatoform and other nonpsychotic mental disorders), 96 (97%) of the Turkish-speaking patients had an ICD-10 diagnosis F40–49. 6 (6.5%) of the Austrian but none of the Turkish-speaking patients had a diagnosis F90–99 (behavioural and emotional disorders with onset usually occurring in childhood and adolescence or unspecified mental disorder; Table 4).

**Table 4:** Patients’ psychiatric diagnosis

ICD-10	Austrian patients (%)	Turkish-speaking patients (%)
*F20–29* Schizophrenia, schizotypal and delusional, and other non-mood psychotic disorders	0	1 (1)
*F30–39* Mood (affective) disorders	2 (2.2)	0
*F40–49* Anxiety, dissociative, stress-related, somatoform and other nonpsychotic mental disorders	80 (87)	96 (97)
*F50–59* Behavioural syndromes associated with physiological disturbances and physical factors	3 (3.3)	1 (1)
*F70–79* Intellectual disabilities	1 (1.1)	1 (1)
*F90–99* Behavioral and emotional disorders with onset usually occurring in childhood and adolescence	6 (6.5)	0
*Data missing*	8	1

Pearson’s chi-square test *p* = 0.047, df = 5; Fisher’s exact test *p* = 0.007

### Comparison of the causes of stress-related disorders and F43 diagnosis according to ICD-10 classification in both study groups

We detected significant differences between the distribution of causes of trauma and ICD-10 diagnosis only for “sexual abuse” (*p* = 0.000^***^) and for “rape” (*p* = 0.038^*^).

Most patients with a diagnosis of F43.1 (post-traumatic stress disorder) were sexually abused or raped.

More Turkish-speaking (78%) than Austrian patients (57.6%) were diagnosed with F43.2 (adaptation disorder). Furthermore, 29 (29.3%) Austrian and 17 (17%) Turkish-speaking patients were diagnosed with F43.1 (post-traumatic stress disorder), while 13 (13.1%) Austrian and 5 (5%) Turkish-speaking patients were diagnosed with F43.0 (acute stress reaction; Table 5).

**Table 5:** The comparison of the causes of stress-related disorders and ICD-10 diagnosis

Causes of stress-related disorders		ICD-10 diagnosis			Pearson’s chi-square test or Fisher’s exact test *p* =
		F43.0 Acute stress reaction (%)	F43.1 Post-traumatic stress disorder (%)	F43.2 Adjustment disorders (%)	
Parent or grandparent death	No	18 (9.9)	39 (21.5)	124 (68.5)	1.0
	Yes	3 (8.6)	8 (22.9)	24 (68.6)	
Parents’ divorce	No	14 (10.9)	27 (20.9)	88 (68.2)	0.769
	Yes	7 (8.0)	20 (23.0)	60 (69.0)	
Parents’ separation	No	15 (12.2)	24 (19.5)	84 (68.3)	0.292
	Yes	6 (6.5)	23 (24.7)	64 (68.8)	
Patient’s separation from parent(s)	No	7 (9.2)	16 (21.1)	53 (69.7)	0.967
	Yes	11 (8.5)	29 (22.3)	90 (69.2)	
Rape	No	21 (10.0)	43 (20.5)	146 (69.5)	0.038
	Yes	0	4 (66.7)	2 (33.3)	
Maltreatment in childhood	No	20 (10.1)	41 (20.7)	137 (69.2)	0.459
	Yes	1 (5.6)	6 (33.3)	11 (61.1)	
Sexual abuse	No	18 (8.9)	39 (19.2)	146 (71.9)	0.000
	Yes	3 (23.1)	8 (61.5)	2 (15.4)	
Neglect	No	16 (8.6)	40 (21.4)	131 (70.1)	0.267
	Yes	5 (17.2)	7 (24.1)	17 (58.6)	
New environment	No	16 (10.3)	36 (23.2)	103 (66.5)	0.580
	Yes	5 (8.2)	11 (18.0)	45 (73.8)	
Conflict in family	No	14 (10.9)	29 (22.7)	85 (66.4)	0.671
	Yes	7 (8.0)	18 (20.5)	63 (71.6)	
Bullying	No	20 (10.5)	40 (21.1)	130 (68.4)	0.567
	Yes	1 (3.8)	7 (26.9)	18 (69.2)	
Sum	No	179	374	1227	–
	Yes	49	141	396	

### Stress-related symptoms in Austrian and Turkish-speaking patients

Table 6 gives an overview of the stress-related symptoms in Austrian and Turkish-speaking patients.

**Table 6:** Stress-related symptoms

Stress-related symptoms	Frequency (%)		Pearson’s χ^2^-test (*p* =)
	Austrian patients	Turkish-speaking patients	
Changed emotional state	83 (83)	88 (88)	0.315
Cognitive disorder	40 (40)	33 (33)	0.304
Perceptual disturbance	12 (12)	18 (18)	0.235
Social withdrawal	35 (35)	31 (31)	0.547
Sleeping problem	49 (49)	29 (29)	0.004
Somatisation	39 (39)	32 (32)	0.301
Behavioural conspicuousness	11 (11)	44 (44)	0.000

We detected significant differences between Austrian and Turkish-speaking patients only for two conditions, “sleeping problems” (*p* = 0.004^**^) and “behavioural problems” (*p* = 0.000^***^). More Austrian than Turkish-speaking patients suffered from sleeping problems, and more Turkish-speaking than Austrian patients exhibited behavioural problems (Table 6).

### Somatic symptoms

Only in one somatic symptom, “losing weight”, were significant differences between Turkish-speaking and Austrian patients detected: significantly more Austrian patients lost weight (*p* = 0.006). The other investigated symptoms, overweight (*p* = 0.138), headache (*p* = 0.323), vomiting (*p* = 0.390), asthma (*p* = 0.306) and bronchitis (*p* = 0.081) were not different between the 2 groups.

### Preferred therapy

As preferred therapy, more Austrian (32.7%) than Turkish-speaking patients (21%) obtained combined psychiatric and psychotherapeutic treatment. More Turkish-speaking (54%) than Austrian (29.6%) patients received only psychiatric treatment (*p* = 0.011).

In all, 68% of the Austrian patients compared to 72% of the Turkish-speaking patients were compliant in the treatment setting.

Psychiatric treatment is to be understood as using pharmacological medication and psychoeducation for managing the disorder. In contrary, psychological or psychotherapeutic treatment is intervening without the use of medication. In both groups the difference in duration of the treatment was not gathered.

## Discussion

The results of the present study show cross-cultural differences in causes, symptoms, psychiatric diagnoses and preferred therapy according to trauma between native Austrians and Turkish-speaking children and adolescents living in Vienna, Austria.

The most frequently observed causes of stressful and traumatic life events in Austrian children and adolescents were divorce or separation of the parents and family conflicts. The divorce rate in the Austrian population is quite high (40.1%) [45]. In our study sample 83.9% of our Austrian patients, but only 45% of the Turkish-speaking patients lived with one of the separated parents.

Significantly, more Austrian than Turkish-speaking patients experienced maltreatment, emotional neglect and bullying. On the other hand, more Turkish-speaking than Austrian patients experienced intra-familiar conflicts as a stressor.

In line with previous studies [46], our results show that broken families and loss of significant others count among the highest risk factors for problem behaviour and psychiatric disorders in children and adolescents. Our data show differences among both study groups in the hierarchy of traumatised events and the handling process [47], as more Turkish-speaking than Austrian patients experienced intra-familiar conflicts as a stressor and traumatic life event. We assume that diverging views and attitudes between parents and their children in Turkish-speaking than Austrian patients are more probable to lead to severe intra-familial conflicts. Being unable to reasonably solve these conflicts often results in psychological problems [48–51]. Although the self-reports of migrant children living in European countries show high levels of ill health and unmet health-care needs [52–55], counselling centres, psychotherapeutic institutions and schools only rarely have culture and language-sensitive offers for adequately advising migrant minors and their families [56].

Nevertheless symptoms of posttraumatic disorders differed in both study groups. Significantly more Austrian than Turkish-speaking patients suffered from sleeping problems and weight loss. On the other hand more Turkish-speaking than Austrian patients presented behavioural problems. Psychosomatic illnesses like being overweight, vomiting, headache, asthma and bronchitis were similarly distributed.

In European countries the adequate treatment of refugees [57] and migrants [31, 35, 41, 58] poses a challenge for mental health services. In 2008, Lindert et al. [59] reported that migrants living in Germany are overrepresented at psychiatric emergency units and that their access to preventive and psychotherapeutic treatment is reduced compared to the native population. Similar to adults, also migrant children living in Europe use most types of healthcare services less than their native peers except for emergency services [58, 60].

However more Turkish-speaking patients than natives were admitted by their families to our psychiatric out-patient clinic. This finding was previously not reported [61] and may be related to the fact that Turkish families receive culture and language-sensitive medical advice at our special outpatient clinic (Out-Patient Clinic for Transcultural Psychiatry and Migration-Induced Disorders in Childhood and Adolescence). Therefore, cultural differences have to be taken into consideration for adequately advising patients with migration background [35]. Otherwise, wrongful diagnoses [62], inappropriate treatment, lack of confidence in therapists, dissatisfaction with the treatment, low compliance and poorer treatment outcomes are to be feared [61]. It is of utmost importance that therapists and physicians have essential knowledge about the cultural background of their young patients in order to treat them adequately. Cultural sensitivity in the treatment is therefore the most important precondition for transcultural competence [37, 60, 63, 64].

In our study sample more Austrian than Turkish-speaking patients were diagnosed with F3 (mood disorders) and F9 (Behavioural and emotional disorders with onset usually occurring in childhood and adolescence) disorders according to ICD-10 classification.

We also found differences in the distribution of F43 (posttraumatic disorders) diagnoses: more Turkish-speaking patients (78%) than Austrian patients (57.6%) were diagnosed with F43.2 (adaptation disorder), and more Austrian (29.3%) than Turkish-speaking patients (17%) were diagnosed with F43.1 (post-traumatic stress disorder), as most of these patients experienced sexual assault, they were sexually abused or raped. In 2008, Biermair [65] reported that children who experienced violence developed more PTSD than children who had other stressors and traumatic life events.

Particularly by treating traumatised children with migration background, psycho-traumatological, religious and cultural aspects have to be considered very cautiously [37, 40, 64]. Migration experience itself may be burdened by traumatising events, especially in forced migration with traumata originating from the situation at home (war, prosecution, violence) [27], during the migration (multiple dangers and threats, separation from the family, sexual and other forms of abuse), and the situation in the host country (economic problems, xenophobic attitudes of the native people, language and acculturation problems) [47]. Consequently diversity-care, the consideration of cultural and religious aspects in treatment, is of utmost importance [57, 66].

In the present study, 54% of Turkish-speaking and 29.6% of native Austrian patients received psychiatric treatment at our outpatient clinic. More Austrian patients obtained in-ward treatment for post-traumatic disorders as well psychiatric, psychological and psychotherapeutic treatment.

Because migrants living in Austria, have an increased risk of poverty [67], we assume that Turkish-speaking patients did not have sufficient means to finance psychological/psychotherapeutic treatment that must be paid by the patients themselves. However, PTSD is one of the stress-related disorders, for which psychotherapy is widely recommended [68]. Psychotherapeutic treatment in Austria is freely available, but unfortunately only to a limited extent; thus access to treatment remains primarily for the wealthier population to which migrants usually do not belong. There are additional limitations related to language problems and the low number of trained trauma therapists with transcultural knowledge for children and adolescents. Even the EU project, “Healthy Inclusion”, in which Austria also participated, came to the conclusion that psychosocial care for migrants is greatly deficient and that the preventive and psychosocial treatment is less available for migrants compared to the native Austrian population [69], the Austrian health-care system virtually does not provide culture and language-sensitive therapy [60, 69, 70].

Moreover our results show that Turkish-speaking patients were more compliant than Austrian patients with therapeutic issues. This may have been caused by a greater confidence of Turkish-speaking families in medical advice at our special transcultural out-patient clinic offering culture and language-sensitive treatment in their mother tongue. Additionally the more family-centred and controlling life-style of Turkish-speaking population increases their attendance and compliance, if they feel themselves confident in the treatment setting.

Although the number of migrants in European countries is large, there are enormous deficits in the culture-specific medical and therapeutic offers. Nevertheless, cultural aspects are quite important for meaning and purpose in life, therefore cultural and transcultural differences should carefully be considered in the medical care of the migrant population [71] to optimize service delivery. In addition, evidence-based studies about migration-related mental-health problems of children and adolescents are rare [72]. To our best knowledge, there is no Austrian study available that compares transcultural differences of traumatised children and adolescents originating from Turkey compared to natives. Our study provides novel and valuable evidence about psychological trauma in the field of transcultural psychiatry of children and adolescents. Furthermore, our findings will support physicians and therapists in the diagnosis, for adequate treatment and care of patients with migration background and different cultural identities. Because the migrant population in Europe is steadily increasing, diversity-care measures are becoming more and more important. The results of this study can especially be used in diversity-care offers, in order to treat children and adolescents with migration background adequately.

## Limitations

Because of the retrospective design of our study, some data on the socio-economic background like family income, parent job, parent financial situation and parent education were missing, which can have an important impact on the treatment of child trauma.

Differences of societal values, socio-economic status, and cultural background are decisive in the process of developing mental health problems [73]. Therefore, it will not be possible to generalize our results for all groups of migrants living in the same or in different host countries.

The available patient reports may have been biased by the subjective view of the patients and their parents and by the pathologizing view of the attending physicians.

In the present study, the symptoms were partly reported by the youths themselves and partly by their parent(s). There may be differences between self and parental reported symptoms, so that the parents did not assess the severity and quality of the symptoms adequately.
